# A community-health partnership response to mitigate the impact of the COVID-19 pandemic on Travellers and Roma in Ireland

**DOI:** 10.1177/1757975921994075

**Published:** 2021-03-03

**Authors:** Jacopo Villani, Petra Daly, Ronnie Fay, Lynsey Kavanagh, Sandra McDonagh, Nurul Amin

**Affiliations:** 1 Mental Health Services, Health Service Executive, Community Healthcare West, Galway, Ireland; 2 Mental Health Services, Health Service Executive, Community Healthcare Midlands Louth Meath, St Lomans Hospital, Mullingar, Ireland; 3 Pavee Point Traveller and Roma Centre, Mountjoy, Dublin, Ireland; 4 Offaly Traveller Movement, Tullamore, Ireland

**Keywords:** primary health care, Irish Travellers, Roma, collaboration/partnerships, pandemic preparedness planning and response, advocacy, health inequities, COVID-19

## Abstract

Irish Travellers and Roma are two ethnic minorities experiencing high levels of health inequities. These communities are at greater risk of developing COVID-19 and of suffering more severe symptoms due to poor living environments and higher rates of comorbidities. This study explores the strategies adopted by community-health partnerships and NGOs to minimise the potential widening of Travellers’ and Roma’s health inequities during the initial response to the COVID-19 pandemic in Ireland. A descriptive qualitative approach was employed to provide a detailed account of three different community and partnership-led responses. Data were gathered from multiple sources and through first-hand participation in the COVID-19 responses. Data were analysed using thematic analysis. This study found that the main pandemic mitigation interventions implemented were public health measures, culturally sensitive communications, lobbying for policy change and economic and social support. These interventions, supported by the health promotion strategies of partnership, advocacy and empowerment, have proven to be extremely important to reduce potential inequities in exposure to the virus and in access to healthcare. The findings suggest that community-health partnerships between minority groups’ organizations and healthcare professionals represent a viable approach to mitigate the disproportionate effects of a pandemic on Travellers and Roma.

## Introduction

In March 2020 the Irish Government introduced a series of public health protection measures to control the COVID-19 pandemic, caused by the severe acute respiratory syndrome coronavirus 2 (hereafter SARS-CoV-2). These included restrictions on non-essential travel, school and workplace closure, social distancing, proper hand washing and quarantining of patient contacts (1). People from low-income and ethnic minority backgrounds may have been disproportionately affected by these measures as they are often unable to work from home, rely on community and school-based services and face multiple barriers to adhere to public health advice (2).

This is indeed the case for Irish Travellers and Roma, two ethnic minority groups whose experience of social marginalisation has been largely documented (3–6). Travellers and Roma marginalisation is attributable to poor access to social and health services, lack of economic and political power and a low level of participation in Irish society (3–6). According to the 2016 national Census (7) and the 2018 *Roma in Ireland—A National Needs Assessment* (6), there are approximately 36,000 Irish Travellers and 5,000 Roma living in Ireland. Statistics show that a considerable proportion of these communities are homeless or live in overcrowded accommodations, and that they experience poorer educational and employment outcomes (4,6,7). It has also been estimated that over 1,000 Travellers are living on the side of the road in trailers (7), and that most Travellers and Roma lack water and sanitation, heating and other basic facilities (4,6). Hence, Travellers’ and Roma’s ability to comply with public health recommendations has been significantly limited by financial insecurity, low literacy rates and sub-standard living environments.

This is compounded by widespread discrimination (4) and the lack of culturally sensitive communication on COVID-19 (5) which may undermine effective community engagement and trust in mainstream health services. A systematic review documented that discrimination and cultural and language barriers are among the most common barriers to accessing healthcare for Travellers and Roma (8).

Studies conducted in Ireland (4) and continental Europe (9) show that Travellers and Roma experience extreme levels of health inequities. This includes a higher prevalence of heart disease, cancer and diabetes which, according to emerging epidemiological evidence on COVID-19 (10), increases susceptibility to the disease and disease severity.

There is evidence of the unequal levels of health outcomes among ethnic minorities during pandemics (11–13), which is corroborated by recent UK data demonstrating excessive COVID-19 death rates among minority groups (14). These findings suggest that Travellers and Roma as marginalised groups are more likely to experience higher levels of risk compared to the general Irish population.

### Conceptual framework

Health inequities are avoidable inequalities in health status between different population groups (15). Whereas ‘health inequality’ simply indicates differences in health which are natural, ‘health inequity’ is charged with a value judgement, referring to differences which are avoidable and unfair. Therefore, tackling health inequities means addressing injustice (15), which is one of the ethical values underpinning health promotion practice.

This study is placed within a health inequity framework which describes the variety of theories on the social production of health and disease (16). While there are different aetiological pathways for the understanding of health inequities, such as cultural/behavioural, psychosocial, selection, life course and material explanations (16), one of the most influential models clarifying how health and disease are socially produced is offered by Diderichsen *et al*. (17).

According to Diderichsen *et al*. (17) people’s social position will determine their 1) exposure to risks (*differential exposure*), it will define their 2) degree of vulnerability to disease if exposed (*differential vulnerability*), and 3) people from different socio-economic groups will suffer different consequences of disease as a result of inequalities in access to treatments (*differential consequences*). As a corollary, unequal levels of health and illness are a direct consequence of social position.

Following Diderichsen *et al*.’s model, which has been applied to pandemic influenza planning in the United States (11), these inequities can be narrowed by decreasing the exposure of disadvantaged people to health damaging factors, by reducing their susceptibility and intervening through the healthcare system to improve timeliness of access to treatments (17).

The literature on pandemic influenza preparedness and response for vulnerable populations suggests that the potential increase of health inequities during pandemics can be avoided by interventions that include culturally appropriate communications, coordination between public health and community safety-net systems, engaging minority organizations and development of partnerships between community-based organizations and healthcare providers (11–13).

Scholarly works have advanced the idea that health promotion principles and strategies can contribute to addressing the challenges of pandemics (18). This includes the strategies of partnership, advocacy and empowerment which were outlined as essential for the pursuit of health in the landmark 1986 Ottawa Charter for Health Promotion (19,20).

While advocacy in health promotion is the process of defending a cause, through empowering disenfranchised groups, or influencing policy makers to achieve public health goals (21), a partnership for health is an alliance of multiple actors aimed at promoting population health (22).

The involvement of communities, minorities and grassroots service users is considered essential in ‘health promoting’ partnerships, because community members can work alongside professionals to influence health among the wider population (22). This type of partnership can foster community engagement and empowerment which is a strategy aimed at enabling people to increase control over their health (19,20). An empowering approach requires the creation of supportive environments, life skills and opportunities, and access to information and appropriate communication (20).

Through the lens of this theoretical framework, this study explores the strategies implemented in Ireland to avoid a disproportionate health impact of COVID-19 on the Traveller and Roma communities.

### The Traveller and Roma health infrastructures in Ireland

In Ireland there are 27 Primary Health Care for Traveller Projects (hereafter PHCTPs) and 3 Roma health projects, which are peer-led initiatives aimed at improving Travellers’ and Roma’s health outcomes through outreach health promotion and education (23,24). These projects employ approximately 300 Traveller community health workers and Roma mediators who use a social determinants of health approach to address health inequities. These government-funded community-based projects are connected to, and monitored by, seven Traveller Health Units (hereafter THUs), which are regional structures under the management of the main state healthcare provider in Ireland: the Health Service Executive (hereafter HSE) (25).

It is against this background that the COVID-19 mitigation plans for Travellers and Roma were implemented.

### Study aims

This research study set out to contribute to the knowledge on pandemic responses for vulnerable populations with new evidence and recommendations from the initial response to the COVID-19 pandemic in Ireland.

The purpose of this study is to explore the contribution of health promotion strategies to minimising the potential exacerbation of Travellers’ and Roma’s health inequities. More specifically, the study examines the wide-ranging interventions implemented by non-governmental organisations (NGOs) and community-health partnerships to limit inequities in exposure to the coronavirus SARS-CoV-2 and in access to healthcare.

This study, therefore, seeks to document effective practices which can inform and improve future policies and programmes in the realm of pandemic preparedness and response for vulnerable ethnic minorities.

## Methods

This study employed a descriptive qualitative approach which, following Sandelowski (26), is suitable for seeking the ‘descriptive validity of events which most people, observing the same phenomena, would agree is accurate’. Through this approach, this study aims to provide a detailed account of three different community and partnership-led responses during Ireland’s first three months of COVID-19 pandemic (March–May 2020).

These responses were selected through purposive sampling, as they are considered representative of the measures implemented in Ireland to mitigate the impact of the pandemic on the Traveller and Roma communities. The authors’ active involvement in the implementation of these responses, and their extensive expertise in working with these populations from community development and healthcare perspectives, has driven the choice for this sampling approach. Although each response is unique and independent there is a small degree of overlap between them, as few interventions initiated at community level by NGOs were supported by partnership responses at a national level.

Researchers gathered evidence first-hand during the COVID-19 responses through participation in interagency meetings, development of advocacy strategies and management of the Traveller COVID-19 helpline. Data were collected from multiple sources such as: NGOs’ briefings, Traveller COVID-19 helpline database, Traveller and Roma-specific COVID-19 multi-media sources, minutes of meetings and observation notes. The data related to each response were collated into three separate folders. Ethical approval was sought and obtained from each group of healthcare providers and NGOs involved in the production of the data used for this study. Anonymity was adhered to throughout the research process and ensured by collecting anonymised information.

Following Braun and Clarke (27), data were analysed using thematic analysis. First, the entire data set was coded through codes derived from the data. In a second phase, some codes were disregarded and others were merged under broader categories (themes). The identification of these categories was mainly conducted through a deductive approach, informed by the literature on pandemic responses and through the lens of health promotion. The data presented in this study were the most prevalent across the data set and the most relevant for the study aims.

## Results

The results of the analysis are presented in Table 1, which shows the different combinations of strategies and interventions adopted by NGOs and community-health partnerships. The mitigation interventions are coded to reflect the four main categories identified from the analysis.

**Table 1.** Strategies and interventions employed to minimise the widening of Traveller and Roma health inequities in Ireland.

*Responses analysed*	*Health promotion strategies employed*	*Mitigation interventions implemented*	*Possible sources of inequity addressed*
Community response in the Eastern region	Advocacy empowerment	Culturally sensitive and literacy friendly communication^a^	Access to healthcare and exposure to the virus
		Distribution of hygiene kits^b^	Exposure to the virus
		Distribution of food, telephones and assistance to access financial support^c^	
		Lobbying for policy changes on accommodations and evictions^d^	
National COVID-19 Traveller and Roma response team	Partnership empowerment advocacy	Lobbying to prioritize Travellers and Roma in COVID-19 testing^d^	Access to healthcare
		Dissemination of translated COVID-19 resources for Roma^a^	Access to healthcare and exposure to the virus
		Provision of isolation facilities for Roma^b^	Exposure to the virus
Traveller COVID-19 helpline	Partnership empowerment	Provision of information on COVID-19, testing and access to healthcare^a^	Access to healthcare and exposure to the virus
		Signposting of vulnerable cases to local Traveller Health Units, PHCTPs and community safety-net systems^c^	Exposure to the virus

a Culturally sensitive communication.

b Public health measures.

c Economic and social support.

d Lobbying for policy change.

Each response will be analysed individually in the following sections. This will show the distinctive features of each response in addressing the intersecting vulnerabilities of the selected populations.

## The community response to the COVID-19 pandemic in the Eastern region

The first community responses to the crisis in the Eastern region were advocacy actions and lobbying initiated by the leading NGO Pavee Point Traveller and Roma Centre (hereafter Pavee Point) and supported by Traveller organizations and HSE partners. Since many Traveller families live in halting sites lacking water and sanitation and other basic facilities, it was paramount to rapidly improve these sites, to enable residents to comply with hygiene advice and protect themselves from COVID-19. Moreover, due to specific Irish legislation which prohibits entering, occupying or bringing any object onto vacant lands (28), many Travellers living in trailers on the side of the road are in breach of such law, and are evicted by law enforcement. Evictions during the pandemic would have increased the vulnerability of a substantial number of Travellers.

In order to tackle these fundamental issues, Pavee Point implemented a set of coordinated activities, such as: writing a briefing paper with key recommendations, meeting with the Minister for Housing and exercising pressure through HSE partners. As a consequence, the Department of Housing issued a national circular to all local authorities taking into consideration Pavee Point’s recommendations and prompting local authorities to improve facilities in halting sites and provide isolation units where necessary (29).

As a direct result of this work, many Traveller halting sites around Ireland have been equipped with extra toilets and waste collection, running water and extra mobile accommodation for isolation. Following a similar course of action, national emergency legislation included a ban on Traveller evictions (29).

At grassroots level, NGOs reported Travellers’ difficulties in understanding key public health messages due to low health literacy and the lack of cultural sensitivity in mainstream communication around COVID-19. As a response NGOs designed culturally sensitive and literacy friendly communications. These can be defined as communications which incorporate ‘the culture (norms, beliefs and values) of the target population’ (30), in order to be more effective and accepted, and consider its literacy needs.

Communication campaigns included the production of video and audio messages, online resources and leaflets, designed *by* Travellers *for* Travellers, on COVID-19 prevention and how to access COVID-19 testing (29). Given the fact that 83% of Travellers receive their health information from the PHCTPs (4), COVID-19 resources were disseminated through Traveller Community Health Workers using social media and mobile phone technology. These workers played a key role in debunking disinformation around COVID-19 and confirming key public health messages.

In addition, many PHCTPs and Traveller groups worked with local agencies to support the distribution of hygiene kits, food, books and telephones to vulnerable Traveller families, and helped them to file applications to access financial support provided by the Red Cross and local charities.

## The community-health partnership responses to COVID-19

The following sections contain an analysis of two partnership initiatives formed at the onset of the crisis.

### The national COVID-19 Traveller and Roma response team

At a national level, a COVID-19 Traveller and Roma Response Team was formed in early March 2020 by the HSE National Social Inclusion Office, whose remit is supporting health access to a number of vulnerable groups (25). This national team included regional THUs and groups working with Roma, members of HSE Public Health, Social Inclusion, Mental Health and Health and Wellbeing. The aim of this group was to share information and data, identify emerging issues at local and regional levels and develop strategic responses.

One of the main achievements of this partnership was the inclusion of Travellers and Roma as vulnerable groups and consequently their prioritisation and fast-tracking for COVID-19 tests. This advocacy result was particularly relevant as these communities are more likely to experience sub-optimal engagement with healthcare services due to literacy, language and cultural barriers (4–6,8). This result was achieved as a result of the information provided by grassroots organizations that documented the challenges faced by the selected populations and built a timely and well-argued case. The long-standing working relationship between NGOs and HSE partners based on mutual trust was conducive to strengthening the alliance and achieving results.

A significant proportion of Roma in Ireland is particularly vulnerable to homelessness and poverty as it does not meet the criteria for the right to reside in the country, which is a prerequisite to accessing social housing and welfare payments (6). This long-standing challenge has been magnified by the COVID-19 pandemic which has exposed Roma to additional health risks. This has been confirmed by the Roma projects and the COVID-19 Roma helpline which reported worrying levels of homelessness and evictions and prompted coordinated actions from the National Response Team.

After weeks of networking and negotiation with different stakeholders, the HSE secured a hotel in the Dublin area for the isolation of COVID-19 positive Roma people from all over the country. This service included transport to the facility, as well as access to medical assistance for the Roma people. In addition, COVID-19 resources and information on how to access public services were translated into different languages for Roma from continental Europe (25), and disseminated through the response team members.

### The Traveller COVID-19 helpline

In recognition of the need for targeted measures to support Travellers during the crisis, a National COVID-19 Traveller helpline was established and managed by the Offaly Traveller Movement in partnership with the THU in Community Healthcare Midlands Louth Meath and HSE Mental Health Service Coordinators for Travellers (31).

The examination of the information collected from 205 calls, received from all over Ireland during 10 weeks of operation, shows that the majority of callers’ concerns were about COVID-19 tests and worry about COVID-19 symptoms. The helpline provided timely information on COVID-19 infection prevention, on the need to quarantine symptomatic individuals and on the pathways to access COVID-19 tests. Through the helpline, Travellers were also advised on community services offering transport to testing facilities, General Practitioner (GP) and hospital appointments.

Concerns related to the social determinants of health represented the second highest reason for calling the service, and more specifically, they were related to overcrowding, food poverty, financial problems, lack of water and sanitation and homelessness. Several callers sought support for the purchase of food and medicines, and disclosed difficulties in managing their weekly budget during lockdown as a result of higher spending on groceries and the loss of income. These economic challenges magnified considerably the psychological impact of the COVID-19 crisis.

Many callers reported high levels of stress caused by fear of infection as a result of the inability to comply with public health advice due to overcrowding and lack of facilities. This included large families living in hotel rooms provided by local authorities as emergency accommodations and young homeless families living in trailers and sharing facilities with their elderly vulnerable parents. The exploration of the helpline database reveals that Travellers’ socio-economic inequities represented a significant source of worry and an obstacle to COVID-19 prevention. This is particularly evident for Traveller women who have been excessively affected by the crisis. Several female callers residing in women’s refuges, and single mothers living in trailers with several children with no running water, toilets and electricity, described with frustration and concern their daily challenges during lockdown.

The helpline was helpful in signposting callers to community services that could collect and deliver meals, essential household items and medication for people in isolation. The helpline was also instrumental in highlighting to the THUs, PHCTPs and local authorities some of the most vulnerable cases in need of support, informing callers on the emergency law protecting Travellers from evictions and helping them to fill out applications for financial support.

## The impact of the pandemic on different gender and age groups

NGOs’ reports revealed that Traveller women have been impacted by a rise in domestic violence during the pandemic. The lack of information about how services operated during the lockdown was a barrier to accessing domestic violence accommodations. In order to improve rates of admission in women’s refuges, Pavee Point developed accessible information for Traveller women that was shared on social platforms.

NGOs reported that Traveller men experienced disproportionate levels of stress during the lockdown. It is plausible that this may have been the consequence of travel restrictions and not being able to provide for their families. In fact, Traveller men, as a result of a very traditional concept of masculinity, associate their identity with earning income and with transport, which is considered essential to generating income (32).

It has been reported that the lack of internet access and suitable hardware threatened Traveller and Roma school children with further educational disadvantage as schools moved to distance learning during lockdown. Pavee Point advocated with the Department of Education for IT support and access to mitigate the effects of school closure.

## Study limitations

Two of the limitations of this study are the use of purposive sampling and the authors’ involvement in the responses analysed, which may have led to the collection of biased data. In addition, the database utilized to log the calls of the Traveller helpline was not designed for research purposes, therefore these data are being used pragmatically and some of the concerns recorded may be subject to different interpretation.

## Discussion

The COVID-19 pandemic demonstrated how the interplay between Traveller and Roma socio-economic vulnerabilities with the challenges posed by the government restrictions threatened to exacerbate existing health inequities by increasing the likelihood of SARS-CoV-2 exposure, susceptibility and delayed access to healthcare (17). The COVID-19 crisis has also exacerbated gender inequities as Traveller women have been disproportionately exposed to risks, especially those living in socioeconomically fragile circumstances.

This study examined the strategies adopted by three community and partnership-led responses to mitigate the impact of the pandemic on Travellers and Roma. The findings suggest that the employment of targeted mitigation interventions, supported by health promotion strategies, contributed to minimise the potential widening of health inequities during the initial response to the pandemic. The data analysed throughout this study shows that the most common approach adopted by NGOs and healthcare providers to achieve equity was to address social inequities experienced by the most disadvantaged families and individuals.

Prior to the pandemic, in Ireland there was an established collaboration between NGOs and healthcare providers through the Traveller Health Units (25). These had been very successful in developing targeted health initiatives for Travellers and Roma. In particular, the Primary Healthcare for Traveller Projects (PHCTPs) has been a successful model of community-oriented healthcare that, for over 20 years, helped to reach out to vulnerable families, compensating for the inability of the health services to deliver appropriate coverage to the whole population (4,23). Notwithstanding the existence of previous partnerships, the threat of a disproportionate health effect on thousands of Travellers and Roma required a closer collaboration with public health specialists and the employment of innovative pandemic mitigation interventions. These included targeted public health measures, economic and social support, culturally appropriate communications and lobbying for policy change (see Table 1). These interventions were supported by the health promotion strategies of partnership, advocacy and empowerment. The partnership approach emerged as being particularly effective in delivering comprehensive pandemic mitigation interventions and advocacy strategies played a pivotal role in achieving rapid policy change.

As shown throughout this article, these actions and strategies intervened to mitigate the processes that generate health inequities as illustrated by Diderichsen *et al*.’s (17) model. The actions implemented to provide economic and social support to affected families have been essential to reducing potential SARS-CoV-2 exposure by enabling adherence to quarantine measures. The employment of lobbying techniques was essential to reduce avoidable inequities in virus exposure through an improvement to living environments and a moratorium on evictions (29). Moreover, lobbying activities reduced possible inequities in access to medical care as a result of the prioritisation of Travellers and Roma for COVID-19 tests.

Culturally sensitive communications and targeted public health measures helped to decrease inequities in access to healthcare and exposure to the virus through provision of isolation facilities, hygiene kits and accessible information on COVID-19 which resonated with Traveller culture’s belief system. This type of communication was particularly important as it has been reported that Travellers consider problems with literacy a major barrier to accessing services and they believe that culturally appropriate information is among the things that would most improve their health (4). Ensuring access to affordable hygiene products and implementing surge plans for isolation units are among the public health and social measures suggested by the World Health Organization in the context of COVID-19 (33).

These actions are in keeping with those recommended for the protection of ethnic minorities during pandemics (11–13). Hence, incorporating these strategies in future pandemic preparedness plans specifically developed for Travellers and Roma would help reduce the unfair and unequal health impact of a pandemic on the most vulnerable.

Pandemic preparedness plans should systematically consider the wider socio-economic needs of Travellers and Roma and should include targeted measures for women, men and children. These should comprise the provision of suitable isolation facilities, improvement of halting site facilities, financial and food support, culturally sensitive communication, fast tracking women and children fleeing domestic violence in social housing allocation, and providing access to internet and hardware for children attending distance learning. Using equity as a guiding principle and a participatory approach to pandemic preparedness planning would ensure that the needs of these communities are accurately reflected (11).

As shown in this study, one of the essential strategies introduced was the establishment of new community-health partnerships at a national level, with a broader range of partners including specialists in public health medicine. These partnerships adopted a successful approach aimed at curbing the spread of the virus by coupling the medical response with a social response and through the synergy of multiple partners from different sectors. This socioecological approach aligns with Kickbusch and Sakellarides’ (18) remarks on the role of health promotion in a pandemic threat and on the importance of focusing on the non-medical measures to control a pandemic.

The partnership-led model was essential for the establishment and management of the Traveller COVID-19 helpline (31). The helpline limited the potential deepening of social and health inequities through the provision of health information and a link to community safety nets to a highly mobile population with low health literacy and poor access to healthcare. Given the positive uptake of this service, establishing helplines for vulnerable populations with no access to the internet would be effective in helping them cope with adversities during a pandemic. Evidence from Greece suggests that a mental health telephone helpline has been crucial during the COVID-19 pandemic to refer emergency cases, network with other services and offer empathetic listening (34).

All the community-health partnerships analysed in this study included representatives of minority ethnic groups and combined NGOs’ knowledge of grassroots needs with the expertise of partners from the healthcare sector. The variety of perspectives and skills combined with a shared commitment to tackle social and health inequities represent, in the view of the authors, a point of strength across these partnerships. These features are consistent with scholars’ suggestions to establish partnerships between public health, NGOs, community based organisations and minority communities to reduce adverse health consequences on ethnic minorities during pandemics (12,13).

The data analysis shows that empowerment is the most prevalent strategy implemented; however, this was mainly limited to the provision of culturally tailored communications and improved living conditions. Although the long-term process of empowerment requires gaining control over the determinants of health (20), it is plausible to believe that the target communities may have moderately increased control over their life choices through greater knowledge of COVID-19 prevention and more supportive environments.

The examination of the community response, and the engagement of the PHCTPs in community health partnerships, demonstrates the value of the primary health care model in supporting disenfranchised communities during a pandemic. Renewed investments in primary health care projects would encourage the creation of a health system that respects cultural needs and protects the right to health of marginalised communities during pandemics.

Finally, socio-epidemiological studies have demonstrated that health inequities are stratified according to social position (17). While structural imbalances cannot be eliminated by pandemic responses and planning, this study has shown that the deepening of health inequities can be minimised during a pandemic by tackling the multiple routes of virus exposure and enhancing access to medical care. This can be achieved through targeted pandemic mitigation interventions delivered through community-health partnerships and the employment of advocacy strategies and empowerment approaches.

## References

[1] Department of the Taoiseach. Speech of an Taoiseach Leo Varadkar TD [Internet]. Dublin: Government Buildings; 2020 3 27 [cited 2021 February 9]. Available from: https://bit.ly/3e4Sqj8

[2] Fortuna LR Tolou-Shams M Robles-Ramamurthy B Porche MV. Inequity and the disproportionate impact of COVID-19 on communities of color in the United States: the need for a trauma-informed social justice response. Psychol Trauma. 2020; 12: 443–445. 32478545 10.1037/tra0000889 PMC8243721

[3] Scullion L Brown P. Understanding the social exclusion of Roma. In: Ahmed A Rogers M (eds). Working with Marginalised Groups: From Policy to Practice. London: Palgrave Macmillan; 2016, pp.70–85.

[4] All Ireland Traveller Health Study Team. All Ireland Traveller Health Study: Our Geels. Dublin: Department of Health and Children; 2010.

[5] Armitage R Nellums LB. COVID-19 and the Gypsy, Roma and Traveller population. Public Health. 2020; 185: 48. 32544696 10.1016/j.puhe.2020.06.003 PMC7275183

[6] Pavee Point Traveller Roma Centre & Department of Justice and Equality. Roma in Ireland—A National Needs Assessment. Dublin: Pavee Point; 2018.

[7] Central Statistics Office. Census Population 2016, Irish Travellers-Demographics [Internet]. Dublin: Central Statistics Office; 2016 [cited 2021 February 9]. Available from: https://bit.ly/2MW49nK

[8] McFadden A Siebelt L Gavine A Atkin K Bell K Innes N, et al. Gypsy, Roma and Traveller access to and engagement with health services: a systematic review. Eur J Public Health. 2018; 28: 74–81. 29346666 10.1093/eurpub/ckx226

[9] La Parra Casado D Gil González D de la Torre Esteve M. The social class gradient in health in Spain and the health status of the Spanish Roma. Ethn Health. 2016; 21: 468–479. 26458079 10.1080/13557858.2015.1093096

[10] Docherty AB Harrison EM Green CA Hardwick HE Pius R Norman L, et al. Features of 20,133 UK patients in hospital with COVID-19 using the ISARIC WHO Clinical Characterisation Protocol: prospective observational cohort study. BMJ. 2020; 369: m1985. DOI: 10.1136/bmj.m1985. 10.1136/bmj.m1985 PMC7243036 32444460

[11] Blumenshine P Reingold A Egerter S Mockenhaupt R Braveman P Marks J. Pandemic influenza planning in the United States from a health disparities perspective. Emerg Infect Dis. 2008; 14: 709–715. 18439350 10.3201/eid1405.071301 PMC2600245

[12] Quinn SC Kumar S Freimuth VS Musa D Casteneda-Angarita N Kidwell K. Racial disparities in exposure, susceptibility, and access to health care in the US H1N1 influenza pandemic. Am J Public Health. 2011; 101: 285–293. 21164098 10.2105/AJPH.2009.188029 PMC3020202

[13] Hutchins SS Fiscella K Levine RS Ompad DC McDonald M. Protection of racial/ethnic minority populations during an influenza pandemic. Am J Public Health. 2009; 99(Suppl 2): S261–S270. 19797739 10.2105/AJPH.2009.161505 PMC4504373

[14] Public Health England. Disparities in the Risk and Outcomes of COVID-19 [Internet]. London: Public Health England; 2020 [cited 2021 February 9]. Available from: https://bit.ly/2yYSQrj

[15] Graham H. Social determinants and their unequal distribution: clarifying policy understandings. Milbank Q. 2004; 82: 101–124. 15016245 10.1111/j.0887-378X.2004.00303.x PMC2690205

[16] Bartley M. Health Inequality: An Introduction to Concepts, Theories and Methods. 2nd ed. Cambridge, MA: Polity Press; 2017.

[17] Solar O Irwin A. A Conceptual Framework for Action on the Social Determinants of Health [Internet]. Geneva: WHO Document Production Services; 2010 [cited 2021 February 9]. Available from: https://www.who.int/sdhconference/resources/ConceptualframeworkforactiononSDH_eng.pdf

[18] Kickbusch I Sakellarides C. Flu city—smart city: applying health promotion principles to a pandemic threat. Health Promot Int. 2006; 21: 85–87. 16714297 10.1093/heapro/dal014

[19] World Health Organization. Ottawa charter for health promotion. Can J Public Health. 1986; 77: 425–430. 3580992

[20] Saan H Wise M. Enable, mediate, advocate. Health Promot Int. 2011; 26(Suppl 2): ii187–ii193. 22080073 10.1093/heapro/dar069

[21] Wise M. The role of advocacy in promoting health. Promot Educ. 2001; 8: 69–74. 11475040 10.1177/102538230100800204

[22] Jones J Barry MM. Factors influencing trust and mistrust in health promotion partnerships. Glob Health Promot. 2018; 25: 16–24. 27466248 10.1177/1757975916656364

[23] McCabe C Keyes F. A Review of Travellers’ Health Using Primary Care as a Model of Good Practice [Internet]. Dublin: Pavee Point Traveller and Roma Centre; 2005 [cited 2021 February 9]. Available from: https://bit.ly/2ALumml

[24] Pavee Point Traveller and Roma Centre. Pavee Point Roma Programme [Internet]. Dublin: Pavee Point Traveller and Roma Centre; 2019 [cited 2021 February 9]. Available from: https://bit.ly/3eeKQlh

[25] HSE National Inclusion Office. Irish Travellers [Internet]. Dublin: HSE National Inclusion Office; 2020 [cited 2021 February 9]. Available from: https://www.hse.ie/eng/about/who/primarycare/socialinclusion/travellers-and-roma/irish-travellers/irish-travellers-information-and-strategy.html

[26] Sandelowski M. Whatever happened to qualitative description? Res Nurs Health. 23: 334–340. 10940958 10.1002/1098-240x(200008)23:4<334::aid-nur9>3.0.co;2-g

[27] Braun V Clarke V. Using thematic analysis in psychology. Qual Res Psychol. 2006; 3: 77–101.

[28] Government of Ireland. Housing (Miscellaneous Provisions) Act [Internet]. 2002. [cited 2021 February 9]. Available from: https://bit.ly/31SRz0k

[29] Pavee Point Traveller and Roma Centre. COVID-19 Information and Resources [Internet]. Dublin: Pavee Point Traveller and Roma Centre; 2020 [cited 2021 February 9]. Available from: https://www.paveepoint.ie/stay-safe-from-coronavirus-covid-19/

[30] Hodge DR Jackson KF Vaughn MG. Culturally sensitive interventions and health and behavioral health youth outcomes: a meta-analytic review. Soc Work Health Care. 2010; 49: 401–423. 20521205 10.1080/00981381003648398

[31] HSE National Inclusion Office. Traveller COVID-19 Helpline [Internet]. Dublin: HSE National Inclusion Office; 2020 [cited 2021 February 9]. Available from: https://bit.ly/2YpF8Yg

[32] Hodgins M Fox F. ‘Causes of causes’: ethnicity and social position as determinants of health inequality in Irish Traveller men. Health Promot Int. 2014; 29: 223–234. 23193193 10.1093/heapro/das066

[33] World Health Organization. Overview of Public Health and Social Measures in the Context of COVID-19-Interim Guidance [Internet]. Geneva: World Health Organization; 18 May 2020 [cited 2021 February 9]. Available from: https://www.who.int/publications-detail-redirect/overview-of-public-health-and-social-measures-in-the-context-of-covid-19

[34] Peppou LE Economou M Skali T Papageorgiou C. From economic crisis to the COVID-19 pandemic crisis: evidence from a mental health helpline in Greece. Eur Arch Psychiatry Clin Neurosci. Epub ahead of print July 2020. DOI: 10.1007/s00406-020-01165-4. PMC7358697 32666279

